# Impact of 2015 earthquakes on a local hospital in Nepal: A prospective hospital-based study

**DOI:** 10.1371/journal.pone.0192076

**Published:** 2018-02-02

**Authors:** Samita Giri, Kari Risnes, Oddvar Uleberg, Tormod Rogne, Sanu Krishna Shrestha, Øystein Petter Nygaard, Rajendra Koju, Erik Solligård

**Affiliations:** 1 Department of Circulation and Medical Imaging, Norwegian University of Science and Technology, Trondheim, Norway; 2 Department of Community Programs, Dhulikhel Hospital, Kathmandu University Hospital, Dhulikhel, Nepal; 3 Clinic of Anaesthesia and Intensive Care, St. Olav’s Hospital, Trondheim University Hospital, Trondheim, Norway; 4 Childrens Clinic, St. Olav’s Hospital, Trondheim University Hospital, Trondheim, Norway; 5 Department of Clinical and Molecular Medicine, Norwegian University of Science and Technology, Trondheim, Norway; 6 Department of Emergency, Dhulikhel Hospital, Kathmandu University Hospital, Dhulikhel, Nepal; 7 Department of Neurosurgery, St. Olav’s Hospital, Trondheim University Hospital, Trondheim, Norway; 8 Institute of Neuroscience, Faculty of Medicine, Norwegian University of Science and Technology, Trondheim, Norway; 9 National Advisory Unit on Spinal Surgery, St. Olav’s Hospital, Trondheim University Hospital, Trondheim, Norway; 10 Department of Internal Medicine, Dhulikhel Hospital, Kathmandu University Hospital, Dhulikhel, Nepal; 11 Mid Norway Sepsis Research Group, Norwegian University of Science and Technology, Trondheim, Norway; National Academy of Medical Sciences, NEPAL

## Abstract

**Introduction:**

Natural disasters pose a great challenge to the health systems and individual health facilities. In low-resource settings, disaster preparedness systems are often limited and not been well described. Two devastating earthquakes hit Nepal within a 17-days period in 2015. This study aims to describe the burden and distribution of emergency cases to a local hospital.

**Methods:**

This is a prospective observational study of patients presenting to a local hospital for a period of 21 days following the earthquake on April 25, 2015. Demographic and clinical information was prospectively registered for all patients in the systematic emergency registry. Systematic telephone interviews were conducted in a random sample of the patients 90 days after admission to the hospital.

**Results:**

A total of 2,003 emergency patients were registered during the period. The average daily number of emergency patients during the first five days was almost five times higher (n = 150) than the pre-incident daily average (n = 35). The majority of injuries were fractures (58%), 348 (56%) in the lower extremities. A total of 345 surgical procedures were performed and the hospital treated 111 patients with severe injuries related to the earthquake (compartment syndrome, crush injury, and internal injury). Among those with follow-up interviews, over 90% reported that they had been severely affected by the earthquakes; complete house damage, living in temporary shelter, or loss of close family member.

**Conclusion:**

The hospital experienced a very high caseload during the first days, and the majority of patients needed orthopaedic services. The proportion of severely injured and in-hospital deaths were relatively low, probably indicating that the most severely injured did not reach the hospital in time. The experiences underline the need for robust and easily available local health services that can respond to disasters.

## Introduction

Earthquakes have a devastating impact on health and the medical infrastructure [1, 2]. Reports from 2006 to 2016 that describe 10 earthquakes worldwide found that between 600 and 220,000 people were killed per event [3]. The number of deaths and injuries vary greatly and depend on factors related to the nature of the disaster and the regional infrastructure. The impact of earthquakes has been reported to be highest in Asia, with China and Pakistan accounting for 40% of all earthquake-related mortality [1]. Following earthquakes in low-resource settings, health facilities are often damaged and thus the emergency response capacity is reduced [1, 4]. A systematic review of earthquakes in developing countries found that lower extremity fractures were the most common type of injury [4].

Nepal has low standard buildings and infrastructure, and is one of the countries in the world that is the most vulnerable to earthquakes [5]. In 2015, Nepal suffered from two earthquakes with magnitudes of 7.8 (April 25^th^) and 7.3 (May 12^th^) on the Richter scale [6, 7]. In total, close to 9,000 people were killed, 22,000 were injured [8], and 2,000,000 people were displaced from their homes [9]. Approximately 90% of the health facilities in the affected areas were destroyed or severely damaged [10]. The functioning health facilities were overwhelmed and there was shortage of medical supplies [11, 12]. As a result, the local health system ability to respond the health care needs in disaster-affected areas was compromised. The international field hospitals functioning three days after the first earthquake reported that only 25% of the patients treated were earthquake-related injuries [13–15].

Disaster preparedness is a key element to resilient health systems [16, 17] and has been emphasized as a global priority from the UN and others [17–19]. Although considerable effort has been devoted to better disaster planning [20], there is still little evidence to support disaster planning and disaster risk reduction activities in low and middle-income countries (LMIC) [17, 21].

Reports have stated that the national health information system of Nepal lack injury details from the earthquakes, and that the national disaster policy was independent to the evidence [12, 22]. Such information is important to develop effective policy, resource allocation and disaster preparedness.

This study aims to describe the emergency patient load and the distribution of earthquake-related injuries (EQIs) and non-earthquake (NEQ) related health problems treated by a local hospital from the first day of earthquake in Nepal in 2015 and the following three weeks. Systematic telephone-interviews were performed in a random sample of these patients 90 days after admission to describe sociodemographic information and the effects that the earthquakes had on this group. We describe the local hospital’s preparedness and share experiences about factors that may be useful for managing disasters in similar settings.

## Materials and methods

### Study design

A prospective observational study of all emergency patients presenting to Dhulikhel Hospital (DH) in Nepal for a period of 21 days after the first earthquake on April 25, 2015, the period include the second earthquake on May 12, 2015.

### Study setting

**Fig 1:**
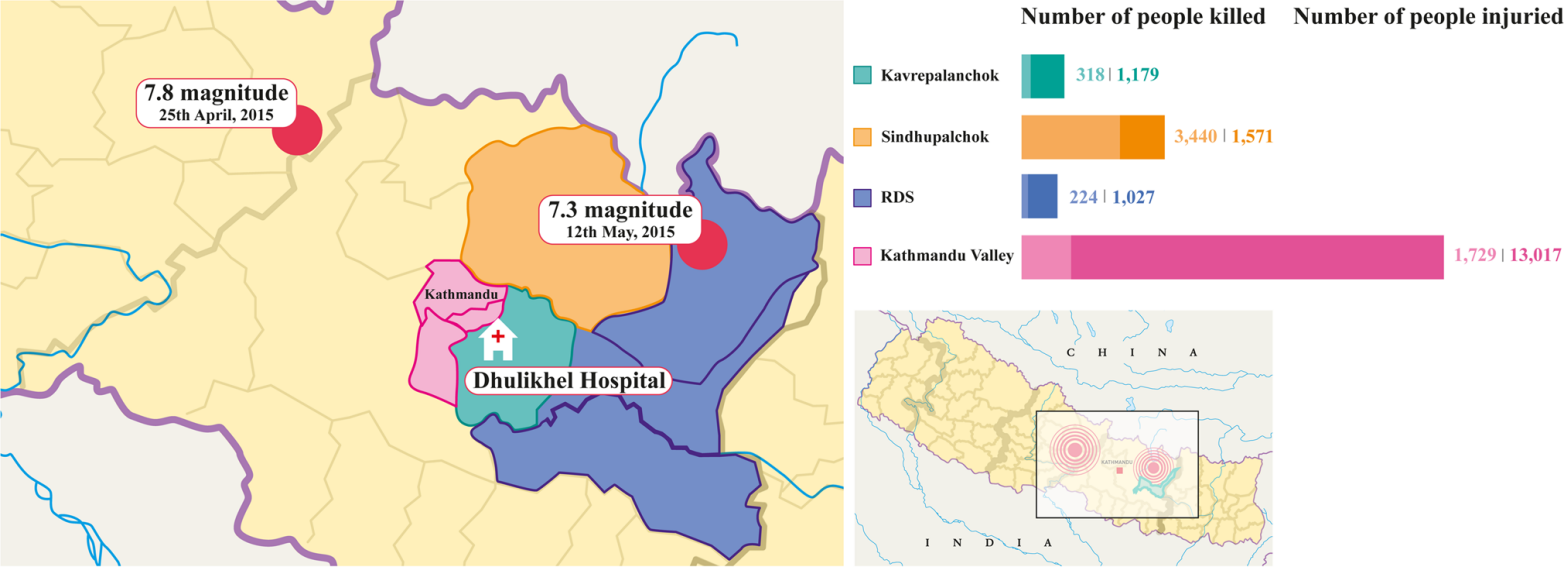
Map of Nepal, Dhulikhel Hospital and its beneficiary districts, including sites of two earthquakes epicenters. Left: enlarged map, illustrating the two earthquakes epicenters (red circles), Dhulikhel Hospital (DH) and its beneficiary districts (coloured sections). Top right bars: the bars show the number of people killed (lighter bar) and injured (darker bar) in the respective DH beneficiary districts [25]. RDS includes the Ramechhap, Dolakha and Sindhuli districts, and Kathmandu valley includes the Kathmandu, Lalitpur and Bhaktapur districts. Bottom right: Map of Nepal showing the two earthquake epicentres.

DH is a non-government university hospital located in the Kavrepalanchok district, which was one of the districts most severely affected by the two earthquakes. DH has a capacity of 375 beds, provides low-cost health services to approximately 1.9 million people from both rural and urban areas and is one of the few specialized hospitals in Kavrepalanchok and the neighbouring districts [23]. DH is located 108 km from the April 25^th^ earthquake epicentre and 84 km from the May 12^th^ earthquake epicentre (Fig 1) [24].

### Dhulikhel Hospital Patient Care project

DH [23] had never been the first-line health care provider after an earthquake. However, the hospital was in the process of improving emergency health care through the “Dhulikhel Hospital Patient Care (DHPCARE)” project, a collaborative project initiated in 2013 between DH, the Norwegian University of Science and Technology (NTNU) and St. Olav’s Hospital, University Hospital Trondheim, Norway [26]. The main interventions in this project were the introduction of a systematic emergency registry, a systematic triage system, and simulator training among health personnel in the emergency department (ED). As part of the project, the ED was reorganized to separate patients into three treatment zones (red, orange/yellow, and green) according to four triage categories (red, orange, yellow and green), with separate staff attending each zone.

### Data collection

Clinical and demographic information was prospectively registered in the systematic emergency registry and hospital patient records for all patients and was extracted for the current study.

### Health-related variables

**Fig 2:**
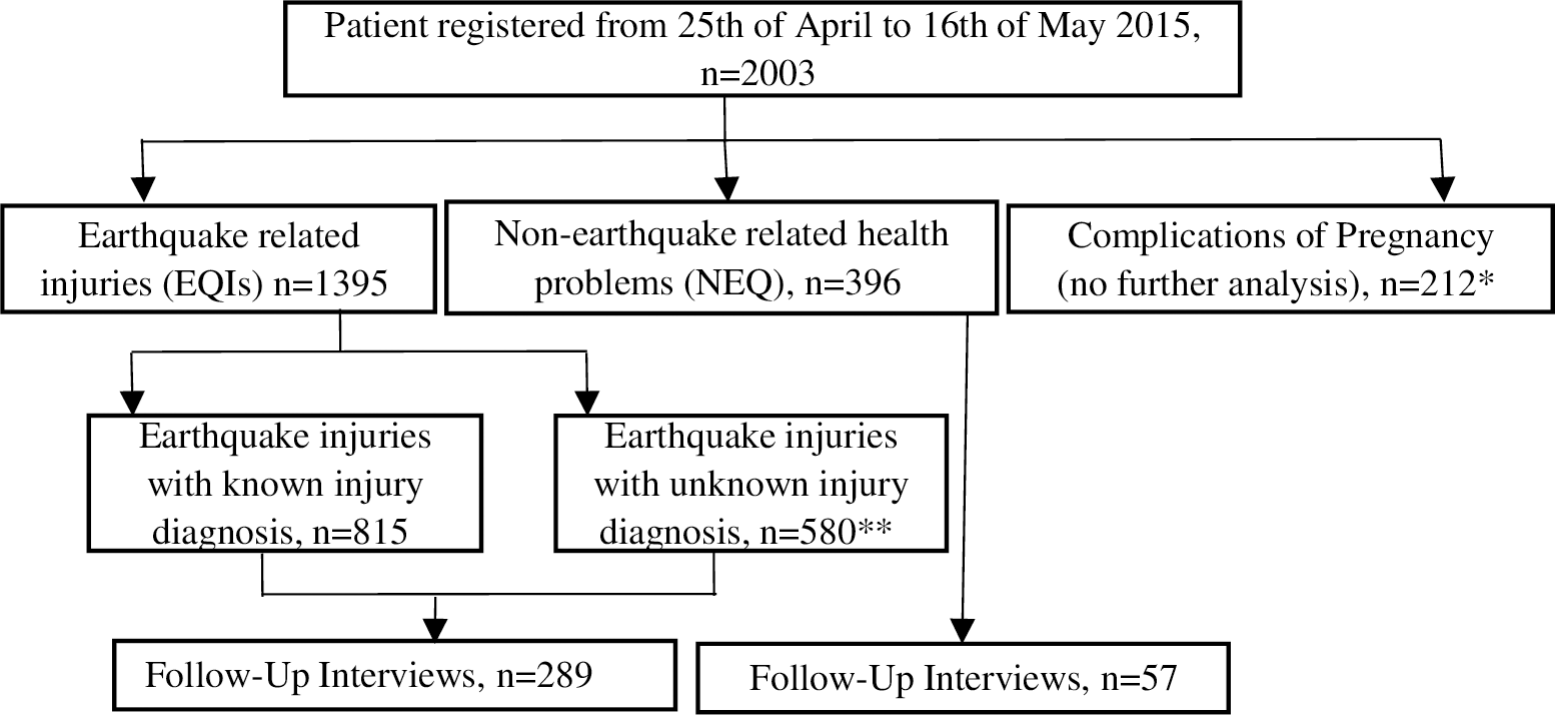
Flowchart of inclusion process. Flowchart of inclusion process of individuals in the study. *Excluded from further analyses because of incomplete information. **Excluded from detailed analyses because of missing information on injury diagnosis.

Patients were classified into three broad categories: Earthquake related injuries (EQI); when the information in the registry included information that injury was caused by earthquake and non-earthquake (NEQ) related health problems; when the information in the registry did not include information that injury or health problem was caused by earthquake (Fig 2). Additionally, any complication of pregnancy (COP) was defined as a separate group. EQIs with documented details on injury diagnoses were further described. The EQIs with known injury diagnoses were categorized into body regions according to the abbreviated injury scale (AIS): head, face, neck/spine, thorax, abdomen, upper extremity, lower extremity and unknown region [27]. There was not sufficient clinical information in order to perform injury grading using the AIS code set [27]. Compartment syndrome, crush injuries and internal injuries were categorized as severe injuries. When the information in the records included only earthquake injury but no details on injury diagnosis, these were classified as earthquake injuries with unknown injury diagnosis.

The NEQ health problems were divided into six categories: infectious diseases, non-communicable disease (NCD), transport accidents, physical assault, poisoning and other NEQ. The categories of ‘other NEQ’ included health problems related to internal medicine, ear nose and throat, gynaecology or psychiatry not otherwise categorized. Mortality that occurred after arrival and during hospitalization was defined as hospital mortality.

### Demographic and geographic variables

We identified four categories of ethnic groups; Brahmin and Chhetri, Janajati, Dalit and other/unknown [28]. Dalit ethnic groups generally have a lower socioeconomic status, whereas Brahmin and Chhetri have a higher socioeconomic status [29]. The patients’ home address was used to categorize their residence into four districts categories: Sindhupalchok, Kavrepalanchok, Kathmandu valley (includes Kathmandu, Bhaktapur and Lalitpur districts) and RDS (includes Ramechhap, Dolakha and Sindhuli districts) (Fig 1). Patients’ arrival time at the hospital was identified by days from the first earthquake on the 25^th^ of April and was categorized into four groups: first week, second week, third week and unknown.

### Follow-up interviews

Among the 1791 included patients for follow-up interviews, a total of 653 patients’ phone numbers were recorded. Of these 653 patients’, 346 patients were randomly selected and were followed up through structured telephone interviews 90 days after admission. Information was collected on health outcome and sociodemographic including the damages from the earthquakes. The impact of the earthquake on their lives were categorized as follows: very severe (loss of any family members or living in a temporary shelter or house completely damaged), moderate (any family members severely injured or migrated to new place or house partly damaged) and minor (any family members with minor injury or return to previous house or minor cracks in house).

### Data analysis

Descriptive statistics using numbers and proportions (%) were calculated. We used 95% confidence intervals (CIs) for percentages in each category to describe the distributions of patients and their characteristics. We used STATA IC 13.1 (StataCorp LP, College Station Texas, USA) for all the analysis.

### Ethics

The study was approved by the institutional review committee of Kathmandu University School of Medical Sciences in Nepal (58/13) and the Regional Committee for Medical and Health Research Ethics in South East Norway (2014/1246). Individual consent for information to be entered the emergency registry was waived by the ethical committee. Because of low literacy in the population, a procedure for oral information and consent was accepted for follow-up interviews and the consent was taken from the legal guardians for the patients under the age 18 years.

## Results

**Fig 3:**
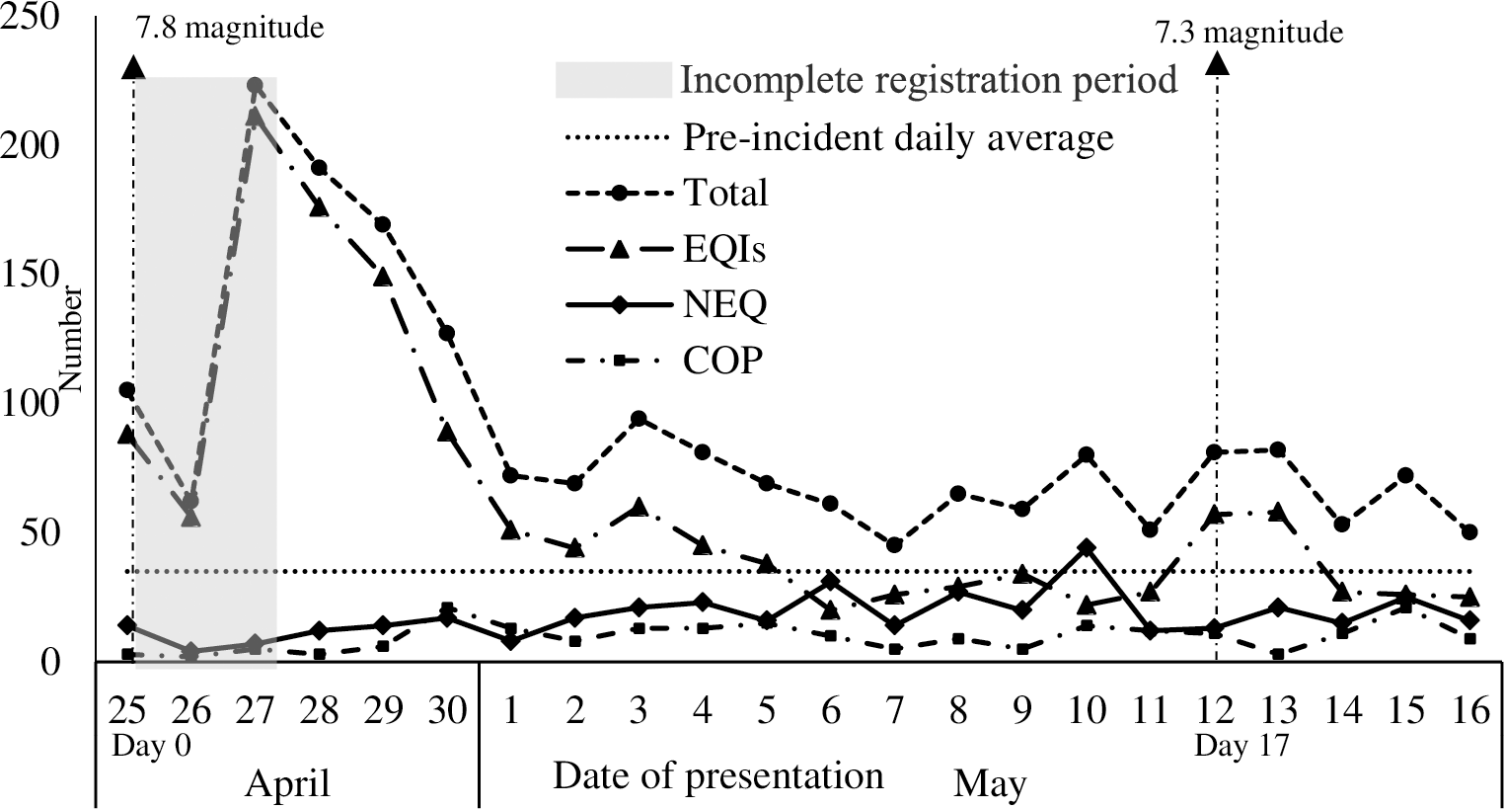
Daily distribution of patients from first earthquake 25^th^ April including second earthquake 12^th^ of May. The horizontal axis refers to the patient presenting days to Dhulikhel Hospital (DH) starting from the first day of earthquake on April 25 (day 0) until day 21 including second earthquake on day 17. The figure shows less number of patients in the first two days but in reality we had overwhelming number of patients but the patient registration system could not be maintained. Number of earthquake injuries was almost five times higher in the first five days compared to pre-incident daily average. The number of patients increased for the first two days after the second earthquake on day 17, indicating the mobile health facilities were in place. NEQ patients increased from day 11 and COP subsequently increased from day 5. EQIs, Earthquake related injuries; NEQ, Non-earthquake related health problems; COP, Complication of pregnancy.

During the study period, we registered 2,003 emergency patients and classified these as shown in Fig 2. Of the 2,003 patients, 1,395 (70%) presented with EQIs, and 396 (20%) with NEQ-related health problems. An additional 212 patients (10%) presented with pregnancy related complications. The distribution of patients during the study period is shown in Fig 3. The total patient load in the hospital in the first five days after the first earthquake was five times higher (n = 150 per day) than the pre-incident daily average (35 emergency patients per day) [26]. The second earthquake occurred on day 17, and the number of EQIs peaked to 80 for the first two days and later returned to the baseline level of approximately 25 patients. Excluding pregnancy complications, the hospital received 896 patients during the first week, 820 (59%) of who were EQIs (Table 1).

**Table 1:** Characteristics of earthquake related injuries and non-earthquake related health problems in Dhulikhel Hospital from 25^th^ April to 16^th^ May 2015.

Demographic Characteristics	Total, n(%)	EQIs, n(%)	95% CI^ǂ^	NEQ, n(%)	95% CI^ǂ^
**Total**	1791	1395 (78)		396 (22)	
**Sex**					
Male	790 (44)	577 (41)	39–44	213 (54)	49–59
Female	1001 (56)	818 (59)	56–61	183 (46)	41–51
**Age (years)**					
<15	326 (18)	252 (18)	16–20	74 (19)	15–23
15–35	563 (31)	459 (33)	30–35	104 (26)	22–31
35–65	659 (37)	513 (37)	34–39	146 (37)	32–42
>65	243 (14)	171 (12)	11–14	72 (18)	15–22
**Median age (IQR)**	35 (19–52)	33 (19–52)		38 (19–55)	
**Ethnicity**					
Janajati^‡^	779 (44)	603 (43)	41–46	176 (44)	40–49
Brahmin & Chhetri^‡‡^	707 (39)	553 (40)	37–42	154 (39)	34–44
Dalit^‡‡‡^	229 (13)	185 (13)	12–15	44 (11)	8–15
Others & Unknown^§^	76 (4)	54 (4)	3–5	22 (6)	4–8
**Arrival Week**					
First Week	896 (50)	820 (59)	56–61	76 (19)	16–23
Second Week	411 (23)	262 (19)	17–21	149 (38)	33–43
Third Week	442 (25)	276 (20)	18–22	166 (42)	37–47
Unknown^§^	42 (2)	37(2)	2–4	5 (1)	1–3
Pre-incident Daily Average	35	-		35	
**District**					
Sindhupalchok^Ϯ^	841 (47)	763 (55)	52–57	78 (20)	16–24
Kavrepalanchok^ϮϮ^	666 (37)	463 (33)	31–36	203 (51)	46–56
Kathmandu Valley^ϮϮϮ^	75 (4)	46 (3)	2–4	29 (7)	5–10
RDS	70 (4)	47 (3)	3–4	23 (6)	4–9
Others & Unknown^§^	139 (8)	76 (6)	4–7	63 (16)	13–20
**Hospital Outcome**					
Admitted	938 (52)	758 (54)	52–57	180 (45)	41–50
Discharged	374 (21)	233 (17)	15–19	141 (36)	31–40
DOR/LAMA*	24 (1)	13 (1)	1–2	11 (3)	2–5
Referred**	56 (3)	20 (1)	1–2	36 (9)	7–12
Dead in hospital***	15 (1)	9 (1)	0.2–1	6 (2)	1–3
Unknown^§^	384 (22)	362 (26)	24–28	22 (5)	4–8
**Length of Hospital Stay, days median(IQR)**	7 (3–16)	8 (3–19)		5 (3–7)	

EQIs, Earthquake related injuries; NEQ, Non-earthquake related health problems; DOR, Discharge on Request; LAMA, Leave against Medical Advice; RDS, Ramechap, Dolakha and Sindhuli districts.

^ǂ^95% confidence interval of the percentages.

^‡^Low ethnic groups.

^‡‡^High ethnic groups.

^‡‡‡^Lowest ethnic groups.

^§^missing information of patients.

^Ϯ^Sindhupalchok is the most affected district by the earthquakes and the nearest place of Sindhupalchok is at least a two-hour drive from the hospital.

^ϮϮ^Hospital district.

^ϮϮϮ^“Kathmandu Valley” includes three districts (Kathmandu, Lalitpur and Bhaktapur).

*24 patients are either discharged on request or left hospital against the medical advice.

**Referred to higher center for higher treatment.

***15 patients died in the hospital during treatment.

The demographic characteristics of the study population are presented in Table 1. The majority of the EQIs were female (n = 818, 59%). Children <15 years accounted for 18% of the EQIs, and the median age of EQIs was 33 years (IQR 19–52). The majority of EQIs were from Sindhupalchok (n = 763, 55%), 33% were from the hospital district (Kavrepalanchok) (n = 463). Most of the EQIs that presented in the emergency room were admitted to the hospital (n = 758, 54%) and they were hospitalized longer than NEQ patients (median 8 versus 5 days). The number of NEQ patients was relatively low during the first 10 days (average 14 per day) but increased after day 10 (average 21 per day), constituting nearly a fourth of the total patient load at day 10 (Fig 2). The most common NEQ health problems were infectious diseases (30%) and NCDs (27%) (S1 Table).

Information on EQIs with known injury diagnosis is presented in Table 2, and more detailed information on fractures is presented in S2 Table. Of the EQIs, 815 (58%) had information on injury diagnoses for a total of 1,083 injuries, 624 (58%) of those were fractures. More than half of the fractures were in the lower extremities (n = 348, 56%). A total of 345 surgical procedures were performed in the operating room on 318 patients, 338 (98%) of these were orthopaedic procedures. The most common procedures were: internal fixations including open and closed reductions (n = 211, 61%), debridement (n = 59, 17%) and external fixation (n = 37, 11%). The hospital treated 111 patients with severe injuries related to the earthquake: compartment syndrome (n = 18), crush injury (n = 36) and internal injury (n = 57). Eight patients with internal head injuries were referred to the hospital in Kathmandu for neurosurgery services. The hospital mortality was low, 1% in EQIs (n = 9) and 2% in NEQ patients (n = 6) (Table 1).

**Table 2:** Type of injuries by body region in 815 earthquake patients with known injury diagnosis presenting to Dhulikhel Hospital from 25^th^ April to 16^th^ May 2015.

Injury Classification	Total, n (%)	Head, n	Face, n	Neck/Spine, n	Thorax, n	Abdomen, n	UE, n	LE, n	Unknown, n
Fracture	624 (58)	6	9	86	20	0	152	348	3
Soft tissue Injury NOS	106 (10)	9	4	2	7	8	18	37	21
Laceration	83 (8)	18	16	0	0	2	16	22	9
Internal^Ϯ^	55 (5)	28	1	7	11	7	1	0	0
Crush	36 (3)	0	0	0	1	1	5	29	0
Compartment	18 (2)	0	0	0	0	0	0	17	1
Contusion	24 (2)	3	0	0	2	5	6	8	0
Dislocation	25 (2)	0	0	1	0	0	3	19	2
Burns	11 (1)	0	0	0	0	0	0	0	11
Sprain	7 (1)	0	0	0	0	0	0	7	0
Avulsion	1 (0.09)	0	0	0	0	0	0	1	0
Degloving	1 (0.09)	0	0	0	0	0	0	1	0
Amputation*	5 (0.4)	0	0	0	0	0	4	1	0
NOS	87 (8)	25	7	2	3	6	14	30	0
**Total**	1083	89	37	98	44	29	219	520	47

UE, Upper Extremity; LE, Lower Extremity; NOS, Not otherwise specified.

Number and percentage of injury types by body region among 815 earthquake patients with a total of 1083 injuries.

^Ϯ^Eight internal head injuries referred to higher-level hospital in Kathmandu.

*Amputation of two lower arm, two fingers and one foot.

A random sample of 346 patients participated in a structured telephone interview 90 days after hospital admission. The majority of the interviewed had poor housing (Kachha) made of mud, bricks, bamboo and timber and majority reported agriculture as their main occupation (Table 3). The earthquake had a severe impact on 91% of the patients’ lives (completely damaged houses or still living in a temporary shelter or loss of any family members). The majority (76%) had been inside their home at the time of the earthquake. The 90-days mortality among EQIs remained low (2%) but was higher in NEQ related patients (11%). The sensitivity analyses showed that the demographic characteristics of the interviewed group did not differ significantly from those of the total group of included patients (S3 Table).

**Table 3:** Characteristics of interviewed patients with earthquake injuries and non-earthquake related health problems treated in Dhulikhel Hospital during a 21 days period from an earthquake.

Demographic Characteristics	Total, n(%)	EQIs, n(%)	95%CI^ǂ^	NEQ, n(%)	95%CI^ǂ^
**Total**	346	289 (84)		57 (16)	
**Sex**					
Male	135 (39)	103 (36)	30–41	32 (56)	43–68
Female	211 (61)	186 (64)	59–70	25 (44)	32–57
**Age**					
<15	48 (14)	44 (15)	12–20	4 (7)	3–17
15–35	121 (35)	103 (36)	30–41	18 (32)	21–45
35–65	136 (39)	111 (38)	33–44	25 (44)	32–57
>65	41 (12)	31 (11)	8–15	10 (17)	10–30
**Ethnicity**					
Janajati	138 (40)	108 (37)	32–43	30 (53)	40–65
Brahmin & Chhetri	163 (47)	144 (50)	44–56	19 (33)	22–47
Dalit	35 (10)	30 (10)	7–14	5 (9)	4–20
Others & Unknown^§^	10 (3)	7 (3)	1–5	3 (5)	3–15
**Occupation**					
Paid Work	74 (22)	57 (20)	16–25	17 (30)	19–43
Agriculture	181 (52)	154 (53)	47–59	27 (47)	35–60
Children & Elderly^Ϯ^	91 (26)	78 (27)	22–32	13 (23)	14–36
**Type of House**^**ᵜ**^					
Concrete *(pakka)*	40 (12)	32 (11)	8–15	8 (14)	7–26
Mud+Concrete *(Kachha+Pakka)*	46 (13)	31 (11)	8–15	15 (26)	16–39
Mud *(Kachha)*	259 (75)	225 (78)	73–82	34 (60)	46–72
**Impact after Earthquake**					
Very Severe*	315 (91)	266 (92)	88–95	49 (86)	74–93
Moderate**	15 (4)	10 (3)	2–6	5 (9)	4–20
Minor***	16 (5)	13 (5)	3–8	3 (5)	2–15
**Location During Earthquake**^**ᵜᵜ**^					
Inside house/office	256 (76)	216 (76)	71–81	40 (73)	59–83
Outside house/office	68 (20)	54 (19)	15–24	14 (25)	16–39
Others	14 (4)	13 (5)	3–8	1 (2)	0.2–12
**Outcome after Discharged**^**ᵜ**^					
Better	308 (89)	263 (92)	87–94	45 (79)	66–20
Unchanged	23 (7)	18 (6)	4–10	5 (9)	4–20
Worse	2 (1)	1 (0.3)	0.04–2	1 (1)	0.02–12
Died^‡^	12 (3)	6 (2)	1–5	6 (11)	5–22

EQIs, Earthquake related injuries; NEQ, Non-earthquake related health problems.

^ǂ^95% confidence interval of the percentages.

^§^missing information in patients records.

^Ϯ^Childrens under age 15 years and elderly more than 65 years.

^ᵜ^One did not respond

^ᵜᵜ^Eight did not respond.

*Very Severe: any family members died or still living in temporary shelter or house completely damaged.

**Moderate: any family members severely injured or migrate to new place or house partly damaged.

***Minor: any family members has minor injuries or back to previous house or house has minor cracks.

^‡^12 patients died after discharged from hospital within 90 days of hospital admission.

## Discussion

We found that the local hospital provided emergency health care to a large number of EQIs, and that the patient load was particularly high during the first five days. The majority of EQIs were female, young and from the severely affected Sindhupalchok district. Most EQIs were lower extremity fractures. The hospital performed orthopaedic procedures on more than 300 of the injured and more than 100 presented with severe injuries. Likely, many lives and much disability was spared because of existing local emergency and surgical services.

### Patient load and the role of local capacity

There are no previous studies describing the patient load at a local hospital in Nepal after the 2015 earthquakes. We found that a very high number of EQIs treated during the first five days. This is in contrast to reports from the international field hospitals that started providing services three days after the first earthquake and reported a relatively low number of EQIs [13–15]. The international medical teams typically need some days after a disaster to initiate their services [13–15]. Until they arrive, patients are treated by the often poorly developed local health system, and many severely injured likely died prior to receiving medical treatment.

The EQI patient load at the hospital was high until 21 days after the first earthquake. This finding is in contrast with a previous study, which found that during the China-Lushan earthquake, the majority (n = 266; 80%) of patients were treated during the first two days [30]. According to the world disaster report in 2007, people in rural mountainous regions had challenges accessing health services [31]. In Nepal, many earthquake affected areas are rural and mountainous and there are continuous landslides [5, 22, 32], which affect transportation and prevent timely access to health facilities. The pre-existing inadequate health system capacity and destruction of the existing health infrastructure in these regions made access to health care even difficult for the affected people [12]. After the second earthquake on May 12, there were fewer EQIs and an increased patient load lasted for the first two days only. This finding could be explained by the fact that many people had moved to a safer place in a temporary shelter after the first earthquake or that many international field hospitals may have been in place by the time of the second earthquake. However, international relief assistance is often based in urban centres and is often not sustainable in a rural setting [31, 33], and we are unaware of whether they reached people in the affected area who were in need. During the response phase, coordination and leadership challenges exist, within and between the healthcare service providers and often a disconnection between a national policy and implementation at a local level especially in LMICs like Nepal [12, 17]. This reflects the importance of strengthening local health systems and developing a better communication and coordination strategy with national and international medical teams. The insufficient development of disaster management likely affected the outcomes of the 2015 earthquakes in Nepal [34].

### Characteristics of EQIs

We found that the majority of EQIs were women (59% versus the 54% proportion of women in the country);[28] this finding may support the conclusion that women were particularly vulnerable to this earthquake, as it occurred on a Saturday morning, when many females would be inside the house busy with household chores. The field hospitals observed similar findings [14], and spinal cord rehabilitation centre reported 56% (n = 65) of spinal injuries were among women [35]. A review study found that, women, young or old people, those with disabilities, having a poor socio-economic status, or being inside the house and having poorly constructed homes had higher health impact during the earthquakes [1, 17, 36]. In the current study, nearly half of the EQIs were under the age of 35 years, and one in five EQIs were children under the age of 15 years. A similar age distribution was found in the field hospital [14]. This finding may be explained by the fact that children are often with their mothers.

The majority of EQIs in DH were from the severely affected Sindhupalchok district [25]. This finding may be surprising, as the closest place to DH in Sindhupalchok is at least a two-hour drive. According to official statistics, this area had a higher number of deaths (n = 3,440) than Kavrepalanchok (n = 318) and more than half of the health facilities including the district hospital were completely damaged [25, 37]. Many severely wounded individuals from this district likely never reached a health facility, and the distance can likely explain the long-lasting peak of EQIs presenting at the hospital and the low mortality and few severe injuries.

### Types, severity and mortality of injuries

Other studies have described that earthquakes typically cause injuries to the musculoskeletal system [38]. This trend was consistent with our findings, which showed that the majority of injuries were fractures, often in lower extremities. In line with our findings, two field hospitals reported that the majority of patients (81% and 44%) had musculoskeletal injuries [13, 14] and that more than half of the injuries were limb fractures [13, 15]. There are also other events comparable to the 2015 earthquakes in Nepal, such as the 2010-Yashu and 2013-Lushan earthquakes in China (magnitude 7.1), which reported fewer lower extremity fractures at 21% and 47%, respectively [30, 36]. In contrast, a study of the 2010-Haiti earthquake (magnitude 7.0) reported a rate of lower extremity fractures of 63% and fewer minor injuries (13%) [13] than our study (20% minor injuries).

More people are killed in earthquakes than in other natural disasters [39]. In the current study, the hospital mortality was low and was comparable with hospital mortality observed during normal days [40]. The proportions of severe injuries such as compartment syndrome, crush injuries and internal injuries were relatively low compared to the total number of injuries. Nevertheless, more than 100 severe injuries were treated in the hospital, and one in three injured patients had at least two different injuries. We believe that many severely injured individuals died before receiving health care because of the undeveloped health care infrastructure, inaccessibility to health facilities and non-functioning transportation systems [41]. The number of patients reaching hospital is not a complete picture of injuries after an earthquake, since many patients were treated by voluntary health workers locally and were not registered. In a review study on earthquake injuries in developing countries, debridement (33%), closed reduction (24%), open reduction and internal fixation (24%) and external fixation (12%) were described as the most common procedures performed [4]. In our study, more than 300 patients had orthopaedic procedure. Internal fixations (open and closed reductions) represented 61% of the performed procedures. Many survivors who reached the hospital in the present study needed treatment for orthopaedic injuries, and these findings are in line with others [4]. Thus, appropriate medical and surgical care capacity is likely to have saved lives and spared disabilities. The high number of internal fixation may also have reduced infectious complications.

### Characteristics of NEQ patients

During disasters, the regular health care for NEQ related health problems is often compromised and disrupted, although acute trauma care is likely jeopardized by inadequately controlled NCDs such as cardiovascular or diabetic disease [7]. A review study in LMICs has emphasized a preparedness need for changing health priorities of the patients during earthquakes, from an acute treatment of injuries to general health needs that is required within the first week [17]. In our study, a relatively low number of patients with NEQ related health problems sought health care in the first week, but the numbers increased to normal levels in the second and third week. From day five, the hospital established an outpatient desk with a medical team to manage NEQ outpatients, and these individuals were not included in the present study. The majority of NEQ health problems were infectious diseases and NCDs, including chronic obstructive pulmonary disease, cardiovascular disease and diabetes. Infectious diseases included gastroenteritis, pneumonia and urinary infections and was higher (30%), compared to pre-incident period previously reported (18%) [42]. A field hospital reported that during this period, 19% of patients had respiratory illness and 17% had a gastrointestinal illness [14]. Twelve patients with severe self-harm poisoning observed in our study, the majority were women. A study from Nepal reported 41% increase in suicides three months after the earthquakes and a field hospital reported 6% of all patients during the earthquake period suffered from psychiatric conditions [14, 43]. Based on our observation of a relatively high number of infections and self-harm, we speculate that infectious diseases and psychiatric issues could be a major problem after disasters such as earthquakes, but the current data cannot fully conclude to this. Thus, treatment interruptions for NEQ patients should be minimized during the acute phase of the emergency, as they will otherwise increase the risk of complications and death.

### Follow-up 90 days after admission

The majority of the interviewed patients were of low socioeconomic status, with poor housing and small-scale agriculture as major source of income. Nearly nine in ten of the interviewed patients had experienced severe damage to their house, had lost a family member, and were still living in a temporary shelter 90 days after the earthquake. These findings are supported by a systematic review, in which socioeconomic status, location of individuals, construction materials and design of the house, emergency response and local health systems were among the risk factors associated with mortality and injury severity during an earthquake [1]. Furthermore, the 90-day mortality of EQIs and NEQ health problems was two times and five times higher than the hospital mortality rate, respectively. Patients who had died at home by 90 days after admission had severe injuries (spinal fracture, blunt trauma), burns or NCDs. Previous studies have not considered mortality after discharge. However, the local tradition is strongly in favour of deaths occurring at home, and severely injured patients could therefore have been discharged to die at home. We are not aware of the follow-up study after earthquakes in similar setting. Post-disaster investigations in a larger sample are needed in the future to further describe health and living conditions in earthquake victims. Nevertheless, the information from the post-discharge interviews reveals that poor people are vulnerable after an earthquake and that the effects of the disaster become severe over time.

### Dhulikhel Hospital experience

In the first day after the earthquake, the number of EQIs escalated in the hospital, where all the beds and almost all the space in the courtyard was occupied and the working conditions were continuously demanding. Power was out most of the time, and internet and telecommunication were down for the first two days. The triage system at the main entrance of the hospital and the three treatment zones (red, orange/yellow, and green) according to four triage codes (red for highly severe, orange, yellow and green for less severe) were very helpful tools in patient management and ensuring timely treatment according to severity. The hospital had a local emergency communication system that was not tele- or web-dependent was important for being able to respond in this situation. The hospital set up immediate medical direction, 24-hour surgical services, infection control teams, and logistical management teams, who had a vital role in managing unexpected workloads and providing efficient and quality health care.

### Limitations

The diagnosis and demographic information was partly incomplete because of the high workload in the hospital during the first days after the first earthquake. We registered many EQIs who did not have detailed information on injury diagnoses and could not be included in the injury description, and their omission may have led to an underestimation to specific injury loads. The diagnoses of the patients was done by different doctors and was often incomplete. To balance this we used the discharge diagnoses and when available we used procedures (CT, X-Ray, cast) and operation information of individual patients. Another limitation is that in the first few days, patients with multiple injuries and head injuries could have been referred to a higher health facility before the registration, which means severe injuries could be underestimated. A high number of women with pregnancy complications presented to the hospital (n = 212) but could not be described in detail because of incomplete information. Patients with NEQ health problems treated in outpatient care that started from day five were not reported in the present study. The follow-up interviews included relatively low proportion of the patients, hindering the ability to draw firm conclusions especially on 90 days mortality. The study included patients treated at only one hospital, and thus the results may not be truly representative at the population level. However, to date we are not aware of any study that has reported the patient burden in a local hospital from 2015 earthquake in Nepal. However, studies with large samples that assess systematic disaster preparedness to evaluate cost-effective interventions in low-resource settings are warranted, and further studies to investigate post-disaster health outcomes, social conditions, disabilities and psychiatric health should be prioritized.

## Final remarks

Most EQIs arrived at the hospital within the first days after the first earthquake, and the local hospital treated a very high number of cases. Thus, the burden of emergency cases was high before the international field hospitals could be established. Our study result underline the importance of developing consistent and robust local health services capable of managing natural disasters such as an earthquake while also maintaining adequate medical care for other patients. The hospital staffs rapidly initiated systematic screening of patients arriving at the hospital using a simplified triage system, and prioritized effective surgical services.

## Supporting information

S1 Table Characteristics of non-earthquake related health problems for patients presenting to Dhulikhel Hospital, Nepal during a 21 day period after an earthquake on 25^th^ April 2015, including a second earthquake on day 17 (12th May 2015). NCD, Non-communicable disease. RDS, includes three districts (Ramechap, Dolakha and Sindhuli). NEQ, Non-earthquake related health problems. Number and percentages of patients’ characteristics among 396 NEQ patients during 21 days of earthquake. ^ǂ^95% CI provided for percentage for each categories. ^Ϯ^Other NEQ, Patients having health problems related to medicine, ear nose throat, surgery, gynecology, neurology and psychiatric. ^§^missing information in the patients records.
(DOCX)

S2 Table Types of fracture in 624 fractures by body region presenting to Dhulikhel Hospital during a 21-day period after an earthquake on 25^th^ April 2015. NOS, Not otherwise specified. Number and percentage of fracture types by body region in 625 fractures.
(DOCX)

S3 Table Characteristics of interviewed and non-interviewed patients that had been treated in Dhulikhel Hospital during a 21-day period after an earthquake. RDS, Ramechap, Dolakha and Sindhuli districts. Number and percentages of patients characteristics among interviewed and non-interviewed. *Interviewed. **Not Interviewed. ^§^missing information in the patients records.
(DOCX)
