# Supplementary material for: Impact of 2015 earthquakes on a local hospital in Nepal: A prospective hospital-based study
Source: PLoS One. 2018 Feb 2;13(2):e0192076. doi: 10.1371/journal.pone.0192076 (PMC5796718; doi:10.1371/journal.pone.0192076)
Supplement: S1 Table — NCD, Non-communicable disease. RDS, includes three districts (Ramechap, Dolakha and Sindhuli). NEQ, Non-earthquake related health problems. Number and percentages of patients’ characteristics among 396 NEQ patients during 21 days of earthquake. ǂ95% CI provided for percentage for each categories. ϮOther NEQ, Patients having health problems related to medicine, ear nose throat, surgery, gynecology, neurology and psychiatric. §missing information in the patients records. (DOCX) [file pone.0192076.s001.docx]

**S1 Table. Characteristics of non-earthquake related health problems for patients presenting to Dhulikhel Hospital, during a 21-day period after an earthquake on 25^th^ April 2015.**

| **Demographic Characteristics** | **Total, n (%)** | **Infectious Diseases, n(%)** | **95%CI^ǂ^** | **NCD, n(%)** | **95%CI^ǂ^** | **Transport Accident, n(%)** | **95%CI^ǂ^** | **Physical Assault, n(%)** | **95%CI^ǂ^** | **Poisoning, n(%)** | **95%CI^ǂ^** | **Other NEQ^Ϯ^, n(%)** | **95%CI^ǂ^** |
| --- | --- | --- | --- | --- | --- | --- | --- | --- | --- | --- | --- | --- | --- |
| **Total** | 396 | 120 (30) |  | 107 (27) |  | 32 (8) |  | 15 (4) |  | 12 (3) |  | 110 (28) |  |
| **Sex** |  |  |  |  |  |  |  |  |  |  |  |  |  |
| Male | 213 (54) | 66 (55) | 46-64 | 57 (53) | 44-63 | 21 (66) | 48-80 | 9 (60) | 34-81 | 4 (33) | 12-64 | 56 (51) | 42-60 |
| Female | 183 (46) | 54 (45) | 36-54 | 50 (47) | 37-56 | 11 (34) | 20-52 | 6 (40) | 19-66 | 8 (67) | 36-88 | 54 (49) | 40-58 |
| **Age (years)** |  |  |  |  |  |  |  |  |  |  |  |  |  |
| <15 | 74 (19) | 39 (33) | 25-41 | 3 (3) | 1-8 | 5 (16) | 7-33 | 2 (13) | 3-42 | 2 (17) | 4-50 | 23 (21) | 14-30 |
| 15-35 | 104 (26) | 38 (32) | 24-41 | 8 (7) | 4-14 | 13 (41) | 25-58 | 6 (40) | 19-66 | 4 (33) | 12-64 | 35 (32) | 24-41 |
| 35-65 | 146 (37) | 25 (21) | 14-29 | 54 (50) | 41-60 | 11 (34) | 20-52 | 6 (40) | 19-66 | 6 (50) | 23-77 | 44 (40) | 31-49 |
| >65 | 72 (18) | 18 (15) | 10-23 | 42 (39) | 30-49 | 3 (9) | 3-26 | 1 (7) | 1-37 | - | - | 8 (7) | 4-14 |
| Median (IQR) |  | 26 (7-46) |  | 60 (45-72) |  | 30 (21-46) |  | 30 (18-40) |  | 34 (18-45) |  | 32 (17-46) |  |
| **Ethnicity** |  |  |  |  |  |  |  |  |  |  |  |  |  |
| Janajati | 176 (44) | 50 (42) | 33-51 | 53 (50) | 40-59 | 14 (44) | 28-61 | 5 (33) | 14-60 | 3 (25) | 8-57 | 51 (46) | 37-56 |
| Brahmin & Chhetri | 154 (39) | 52 (43) | 35-52 | 37 (34) | 26-44 | 12 (38) | 22-55 | 7 (47) | 23-71 | 5 (42) | 18-70 | 41 (37) | 29-47 |
| Dalit | 44 (11) | 10 (8) | 5-15 | 15 (14) | 9-22 | 3 (9) | 3-26 | 2 (13) | 3-42 | 3 (25) | 8-57 | 11 (10) | 6-17 |
| Others & Unknown^§^ | 22 (6) | 8 (7) | 3-13 | 2 (2) | 0.4-7 | 3 (9) | 3-26 | 1 (7) | 1-37 | 1 (8) | 1-44 | 7 (6) | 3-13 |
| **Arrival Week** |  |  |  |  |  |  |  |  |  |  |  |  |  |
| First Week | 76 (19) | 31 (26) | 19-34 | 14 (13) | 8-21 | 6 (19) | 9-36 | 2 (13) | 3-42 | 3 (25) | 8-57 | 20 (18) | 12-27 |
| Second Week | 149 (38) | 40 (33) | 25-42 | 49 (46) | 37-55 | 12 (37) | 22-55 | 7 (47) | 23-71 | 6 (50) | 23-77 | 35 (32) | 24-41 |
| Third Week | 166 (42) | 49 (41) | 32-50 | 44 (41) | 32-51 | 12 (37) | 22-55 | 6 (40) | 19-66 | 3 (25) | 8-57 | 52 (47) | 38-57 |
| Unknown^§^ | 5 (1) | - | - | - | - | 2 (6) | 2-22 | - | - | - | - | 3 (3) | 1-8 |
| **District** |  |  |  |  |  |  |  |  |  |  |  |  |  |
| Sindhupalchok | 78 (20) | 22 (18) | 12-26 | 25 (23) | 16-32 | 3 (9) | 3-26 | 1 (7) | 1-37 | 1 (8) | 1-44 | 26 (24) | 17-33 |
| Kavrepalanchok | 203 (51) | 64 (53) | 44-62 | 57 (53) | 44-63 | 15 (47) | 30-64 | 10 (66) | 40-86 | 8 (67) | 36-88 | 49 (45) | 35-54 |
| Kathmandu Valley | 29 (7) | 8 (7) | 3-13 | 5 (5) | 2-11 | 4 (13) | 5-29 | - | - | - | - | 12 (11) | 6-18 |
| RDS | 23 (6) | 8 (7) | 3-13 | 4 (4) | 1-10 | 3 (9) | 3-26 | 1 (7) | 1-37 | 1 (8) | 1-44 | 6 (5) | 2-12 |
| Others & Unknown^§^ | 63 (16) | 18 (15) | 10-23 | 16 (15) | 9-23 | 7 (22) | 11-40 | 3 (20) | 6-48 | 2 (17) | 4-50 | 17 (15) | 10-24 |
| **Death** | 6 (2) | 2 (2) |  | 3 (3) |  | - |  | - |  | - |  | 1 (1) |  |

NCD, Non-communicable disease. RDS, includes three districts (Ramechap, Dolakha and Sindhuli)

Number and percentages of patients characteristics among 396 NEQ patients during 21 days of earthquake. ^ǂ^95% CI provided for percentage for each categories. ^Ϯ^Other NEQ, Patients having health problems related to medicine, ear nose throat, surgery, gynecology, neurology and psychiatric. ^§^missing information in the patients records
