# Supplementary material for: Impact of 2015 earthquakes on a local hospital in Nepal: A prospective hospital-based study
Source: PLoS One. 2018 Feb 2;13(2):e0192076. doi: 10.1371/journal.pone.0192076 (PMC5796718; doi:10.1371/journal.pone.0192076)
Supplement: S2 Table — NOS, Not otherwise specified. Number and percentage of fracture types by body region in 625 fractures. (DOCX) [file pone.0192076.s002.docx]

**S2 Table. Types of fracture in 624 fractures by body region presenting to Dhulikhel Hospital during a 21-day period after an earthquake on 25^th^ April 2015.**

| **Body region** | **Type of Fracture** | **Number** | **Total, n(%)** |
| --- | --- | --- | --- |
| Head | Skull | 6 | 6 (1) |
| Face | Nose  Orbit  Zygoma  Mandible | 2  2  3  2 | 9 (1) |
| Neck/Spine | Cervical  Thoracic  Lumbar  NOS | 2  24  53  7 | 86 (14) |
| Thorax | Rib | 20 | 20 (3) |
| Upper extremity | Clavicle  Scapula  Humerus  Radius  Ulna  Hand  Finger  NOS | 11  3  48  33  24  17  14  2 | 152 (24) |
| Lower extremity | Pelvis  Femur  Patella  Tibia  Fibula  Calcaneus  Foot  NOS | 44  96  2  126  45  12  11  12 | 348 (56) |
| Unknown region | NOS | 3 | 3 (0.4) |
| **Total** |  |  | 624 |

NOS, Not otherwise specified
