# Supplementary material for: Impact of 2015 earthquakes on a local hospital in Nepal: A prospective hospital-based study
Source: PLoS One. 2018 Feb 2;13(2):e0192076. doi: 10.1371/journal.pone.0192076 (PMC5796718; doi:10.1371/journal.pone.0192076)
Supplement: S3 Table — RDS, Ramechap, Dolakha and Sindhuli districts. Number and percentages of patients characteristics among interviewed and non-interviewed. *Interviewed. **Not Interviewed. §missing information in the patients records. (DOCX) [file pone.0192076.s003.docx]

**S3 Table. Characteristics of interviewed and non-interviewed patients that had been treated in Dhulikhel Hospital during a 21-day period after an earthquake.**

| **Demographic Characteristics** | **Total,n(%)** | **Int^*^, n(%)** | **95%CI^ǂ^** | **No Int^**^, n(%)** | **95%CI^ǂ^** |
| --- | --- | --- | --- | --- | --- |
| **Total** | 1791 | 346 (19) |  | 1445 (81) |  |
| **Sex** |  |  |  |  |  |
| Male | 790 (44) | 135 (39) | 34-44 | 655 (45) | 43-48 |
| Female | 1001 (56) | 211 (61) | 56-66 | 790 (55) | 52-57 |
| **Age** |  |  |  |  |  |
| <15 | 326 (18) | 48 (14) | 11-18 | 278 (19) | 17-21 |
| 15-35 | 563 (31) | 121 (35) | 30-40 | 442 (31) | 28-33 |
| 35-65 | 659 (37) | 136 (39) | 34-45 | 523 (36) | 34-39 |
| >65 | 243 (14) | 41 (12) | 9-16 | 202 (14) | 12-16 |
| **Ethnicity** |  |  |  |  |  |
| Janajati | 779 (44) | 138 (40) | 35-45 | 641 (44) | 42-47 |
| Brahmin & Chhetri | 707 (39) | 163 (47) | 41-52 | 544 (38) | 35-40 |
| Dalit | 229 (13) | 35 (10) | 7-14 | 194 (13) | 12-15 |
| Others & Unknown^§^ | 76 (4) | 10 (3) | 2-5 | 66 (5) | 4-6 |
| **Arrival Week** |  |  |  |  |  |
| First Week | 896 (50) | 186 (54) | 48-59 | 710 (49) | 47-52 |
| Second Week | 411 (23) | 71 (21) | 17-25 | 340 (24) | 21-26 |
| Third Week | 442 (25) | 88 (25) | 21-30 | 354 (25) | 22-27 |
| Unknown^§^ | 42 (2) | 1 (0.2) | 0-2 | 41 (3) | 2-4 |
| **District** |  |  |  |  |  |
| Sindhupalchok | 841 (47) | 167 (48) | 43-54 | 674 (47) | 44-49 |
| Kavrepalanchok | 666 (37) | 145 (42) | 37-47 | 521 (36) | 34-39 |
| Kathmandu Valley | 75 (4) | 11 (3) | 2-6 | 64 (4) | 3-6 |
| RDS | 70 (4) | 18 (5) | 3-8 | 52 (4) | 3-5 |
| Others & Unknown^§^ | 139 (8) | 5 (1) | 1-3 | 134 (9) | 8-11 |

RDS, Ramechap, Dolakha and Sindhuli districts.

Number and percentages of patients characteristics among interviewed and non-interviewed. ^*^Interviewed. ^**^Not Interviewed. ^ǂ^95% confidence interval for the percentages. ^§^missing information in the patients records.
